# Central stress processing, T-cell responsivity to stress hormones and disease severity in multiple sclerosis

**DOI:** 10.1093/braincomms/fcac086

**Published:** 2022-04-04

**Authors:** Jelena Brasanac, Stefan Hetzer, Susanna Asseyer, Joseph Kuchling, Judith Bellmann-Strobl, Kristin Ritter, Stefanie Gamradt, Michael Scheel, John-Dylan Haynes, Alexander U. Brandt, Friedemann Paul, Stefan M. Gold, Martin Weygandt

**Affiliations:** 1 Charité—Universitätsmedizin Berlin, Corporate Member of Freie Universität Berlin, Humboldt-Universität zu Berlin, and Berlin Institute of Health, NeuroCure Clinical Research Center, 10117 Berlin, Germany; 2 Department of Psychiatry and Psychotherapy, Charité—Universitätsmedizin Berlin, Corporate Member of Freie Universität Berlin, Humboldt-Universität zu Berlin, and Berlin Institute of Health, Campus Benjamin Franklin, 12203 Berlin, Germany; 3 Experimental and Clinical Research Center, a Cooperation between the Max Delbrück Center for Molecular Medicine in the Helmholtz Association and Charité Universitätsmedizin Berlin, Berlin, Germany; 4 Charité—Universitätsmedizin Berlin, Corporate Member of Freie Universität Berlin and Humboldt-Universität zu Berlin, Experimental and Clinical Research Center, Lindenberger Weg 80, 13125 Berlin, Germany; 5 Max Delbrück Center for Molecular Medicine in the Helmholtz Association (MDC), Berlin, Germany; 6 Charité—Universitätsmedizin Berlin, Corporate Member of Freie Universität Berlin, Humboldt-Universität zu Berlin, and Berlin Institute of Health, Berlin Center for Advanced Neuroimaging, 10117 Berlin, Germany; 7 Department of Neurology, Charité—Universitätsmedizin Berlin, Corporate member of Freie Universität Berlin, Humboldt-Universität zu Berlin, and Berlin Institute of Health, 10117 Berlin, Germany; 8 Berlin Institute of Health (BIH), 10178 Berlin, Germany; 9 Department of Neuroradiology, Charité—Universitätsmedizin Berlin, Corporate member of Freie Universität Berlin, Humboldt-Universität zu Berlin, and Berlin Institute of Health, 10117 Berlin, Germany; 10 Charité—Universitätsmedizin Berlin, Corporate Member of Freie Universität Berlin, Humboldt-Universität zu Berlin, Berlin Institute of Health, Bernstein Center for Computational Neuroscience, 10117 Berlin, Germany; 11 Department of Neurology, University of California, Irvine, CA, USA; 12 Department of Psychosomatic Medicine, Charité—Universitätsmedizin Berlin, Corporate member of Freie Universität Berlin, Humboldt-Universität zu Berlin, and Berlin Institute of Health, 10117 Berlin, Germany; 13 Institute of Neuroimmunology and Multiple Sclerosis (INIMS), Center for Molecular Neurobiology Hamburg, Universitätsklinikum Hamburg-Eppendorf, 20251 Hamburg, Germany

**Keywords:** multiple sclerosis, psychological stress, T-cell glucocorticoid signalling, functional brain connectivity, arterial spin labelling functional MRI

## Abstract

Epidemiological, clinical and neuroscientific studies support a link between psychobiological stress and multiple sclerosis. Neuroimaging suggests that blunted central stress processing goes along with higher multiple sclerosis severity, neuroendocrine studies suggest that blunted immune system sensitivity to stress hormones is linked to stronger neuroinflammation. Until now, however, no effort has been made to elucidate whether central stress processing and immune system sensitivity to stress hormones are related in a disease-specific fashion, and if so, whether this relation is clinically meaningful. Consequently, we conducted two functional MRI analyses based on a total of 39 persons with multiple sclerosis and 25 healthy persons. Motivated by findings of an altered interplay between neuroendocrine stress processing and T-cell glucocorticoid sensitivity in multiple sclerosis, we searched for neural networks whose stress task-evoked activity is differentially linked to peripheral T-cell glucocorticoid signalling in patients versus healthy persons as a potential indicator of disease-specific CNS–immune crosstalk. Subsequently, we tested whether this activity is simultaneously related to disease severity. We found that activity of a network comprising right anterior insula, right fusiform gyrus, left midcingulate and lingual gyrus was differentially coupled to T-cell glucocorticoid signalling across groups. This network’s activity was simultaneously linked to patients’ lesion volume, clinical disability and information-processing speed. Complementary analyses revealed that T-cell glucocorticoid signalling was not directly linked to disease severity. Our findings show that alterations in the coupling between central stress processing and T-cell stress hormone sensitivity are related to key severity measures of multiple sclerosis.

## Teaser

Brasanac *et al*. report that the link between stress-induced brain activity and stress hormone T-cell sensitivity in multiple sclerosis patients differed from that in controls. Simultaneously, this activity was linked to patients’ disease severity. This might suggest that an altered CNS–immune system crosstalk in multiple sclerosis is clinically meaningful.

## Graphical Abstract

**Graphical Abstract:**
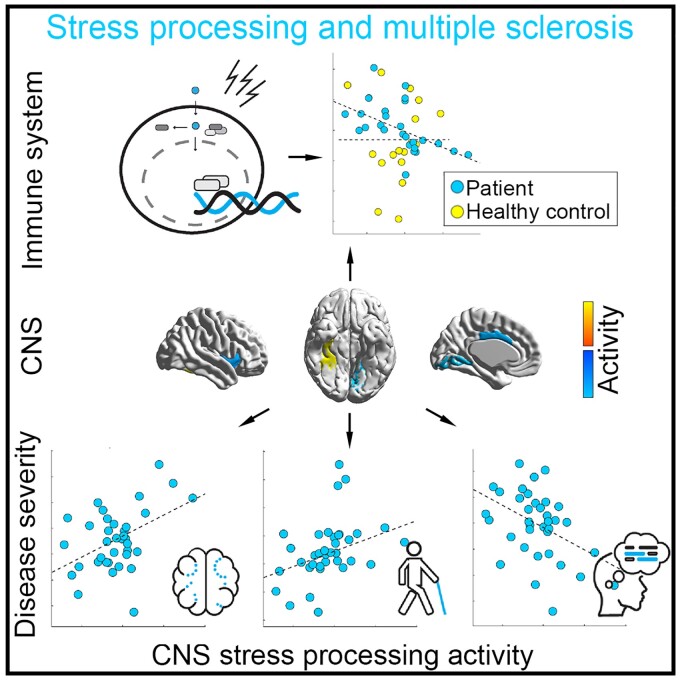

## Introduction

Multiple sclerosis is a demyelinating disease of the CNS characterized by neuroinflammation and neurodegeneration.^1^ The idea that stress might contribute to triggering multiple sclerosis attacks can be traced back to the first description of the disease by Charcot.^2^ Similarly, persons with multiple sclerosis (PwMS) report that they perceive psychological stress as one of the leading triggers for relapses.^3^ This notion was also corroborated in a large population and sibling matched retrospective cohort study indicating that a diagnosis of stress-related disorders significantly increased the risk of subsequently developing an autoimmune disease, including multiple sclerosis.^4^ Prospective and retrospective cohort studies demonstrated an elevated risk of relapses during times of increased stress (ranging from daily hassles to severe life events), as confirmed by a meta-analysis.^5^ The most dramatic examples of such associations were reported in multiple sclerosis centers in Israel and Lebanon, where relapse rates increased strongly during military hostilities.^6,7^ Finally, participation in a stress management programme reduced the incidence of new lesions in a follow-up period in a randomized controlled trial.^8^

Neuroscience has shed light on peripheral, neuroendocrine, and central contributors to stress processing. The hypothalamic–pituitary–adrenal (HPA) axis and the autonomic nervous system (ANS) are regulators of the key peripheral stress hormones cortisol (HPA) and (nor-) adrenaline (ANS) and both, the HPA and ANS receive regulatory inputs from higher-level brain regions including prefrontal, (para-) limbic and cerebellar regions.^9^ Consistently, the activity of these regions^10–12^ and their interplay or functional connectivity (FC), respectively, have been identified as correlates of stress processing.^13–16^ From a clinical standpoint, FC might be especially important as the impact of stress can be affected by coping strategies such as emotion regulation,^17,18^ which is primarily reflected by the interplay between regions.^19^

Until now, biological mechanisms potentially mediating the association between multiple sclerosis and stress were investigated in an isolated, discipline-specific fashion. Neuroendocrine studies found that impaired regulation of the HPA axis is linked to disease severity.^20,21^ Furthermore, they found that PwMS has reduced sensitivity to stress hormone regulation in T cells,^22–24^ which may contribute to neuroinflammation. Neuroimaging studies investigated potential mechanisms of disease severity from a systems neuroscience perspective and found that blunted neural stress processing in the anterior insula (AI), a key interface between neural and immune functions,^25^ is associated with higher clinical disability in a cross-sectional study.^10^ Additionally, we showed that blunted neural stress processing in limbic brain networks is linked to heightened future brain atrophy accumulated across roughly 1000 days in PwMS in a longitudinal study.^26^ Thus, although these studies provided first insights into the potential contribution of individual stress (-related) mechanisms, they did not investigate their interplay.

Consequently, we conducted a study employing an fMRI stress paradigm comprising mental arithmetic with feedback to measure stress-induced alterations in FC reflected by alterations in neural network activity. Additionally, we measured glucocorticoid (GC)-related gene expression in CD4+ and CD8+ T cells as a cellular measure of immune system responsivity to stress hormones. We aimed at answering two key questions. Motivated by findings demonstrating CNS regulation of peripheral inflammation in healthy persons (HPs),^27^ impairment of this regulation in stroke^28^ and the impaired interplay between neuroendocrine stress processing and T-cell GC sensitivity in multiple sclerosis,^22–24^ we first asked whether an altered CNS–immune system crosstalk exists in multiple sclerosis. We tested the corresponding hypothesis (H1) by evaluating whether neural networks exist, whose stress-induced activity is differentially linked to GC-related T-cell gene expression in both groups. Second, we asked whether such an altered CNS–immune system interplay is also clinically meaningful. Following studies underlining the importance of psychobiological stress processing for heterogenous multiple sclerosis severity measures, we thus hypothesized and tested associations between the activity of (i) network(s) fulfilling H1 and four important disease severity measures, i.e. grey matter (GM) fraction (H2^21^), T_2_-weighted lesion load (H3^8^), clinical disability (H4^20^) and information-processing speed (H5^29^).

## Materials and methods

### Participants

Forty-five persons with relapsing-remitting multiple sclerosis (RRMS) were recruited via the Charité outpatient clinics of the NeuroCure Clinical Research Center and the Experimental and Clinical Research Center. Thirty HPs were recruited by advertisements and newsletters. Recruitment and data collection took place between May 2017 and December 2018. Brain imaging took place in the Berlin Center for Advanced Neuroimaging. All participants provided written informed consent before enrolment and received financial reimbursement for their time and effort. Patients visited the outpatient/brain imaging centre for 2 days within a 2-week period (Day 1: clinical assessments and blood draws, Day 2: neuroimaging).

Inclusion criteria for patients were (i) meeting diagnostic criteria for RRMS^30^; (ii) stable treatment with immunomodulatory drugs for the last 3 months [or no disease-modifying treatment (DMT)]; (iii) age ≥18 years; (iv) the physical and mental capability to use the test devices without restriction. Patients were excluded if they (i) were pregnant; (ii) met diagnostic criteria for psychiatric disorders (other than affective disorders including major depression or anxiety disorders); (iii) had a diagnosis or history of neurological disorders (other than multiple sclerosis); (iv) multiple sclerosis relapse or steroid treatment in the last 4 weeks; or (v) had contraindications for MRI scanning. Except for RRMS diagnosis, relapses and treatments, the inclusion and exclusion criteria were the same for controls.

After the application of inclusion and exclusion criteria, the sample comprised 66 participants. Following quality assurance steps (preprocessing), 64 had high-quality data for either fMRI or gene expression (*N*_MS_ = 39; *N*_HP_ = 25). This data set serves as a reference for characterizing clinico-demographic sample characteristics. Please see individual analyses for further information on respective sample sizes. The sample size is highly compatible with the sample size in our recent study investigating associations between central stress processing and disease severity measures in multiple sclerosis irrespective of a potential interplay with T-cell GC sensitivity based on independent data set.^10^ The study was conducted in accordance with relevant guidelines (Helsinki Declaration of 1975) and approved by the ethics committee of Charité—Universitätsmedizin Berlin (EA1/208/16).

### Clinical assessments

Patients underwent neurological examination by experienced neurologists (J.B., F.P., S.A., J.K.). Clinical disability was assessed using Expanded Disability Status Scale (EDSS) and its functional subscales.^31^ All participants underwent a physical exam including a screening test for cognitive function/information-processing speed [Symbol Digit Modalities Test (SDMT)^32^]. Additionally, participants completed the Beck-Depression Inventory (BDI-I^33^).

### Experimental stress paradigm

We employed a widely established arterial spin labelling (ASL) fMRI stress task comprising mental arithmetic and performance feedback that corresponds to a shortened version of the one used in our work,^10,26^ which was derived from previous studies.^12,34^ The paradigm comprised five consecutive stages. In three rating periods (Stages 1, 3 and 5), the participants reported their current degree of feeling stressed, relaxed, anxious and frustrated on a nine-point scale ranging from ‘not at all’ to ‘strongly’ with an MR-compatible response box (please note that only the stress ratings were evaluated in this work). Each of these stages had a duration of 2 min. In the second stage (‘2. Rest’; 8 min), resting ASL fMRI scans and heart rate signals were acquired (see Supplementary material for details on heart rate data processing). The fourth stage (‘4. Stress’; 12 min) comprised the fMRI stress task. Pulse data were acquired in parallel to brain activity.

**Figure 1:**
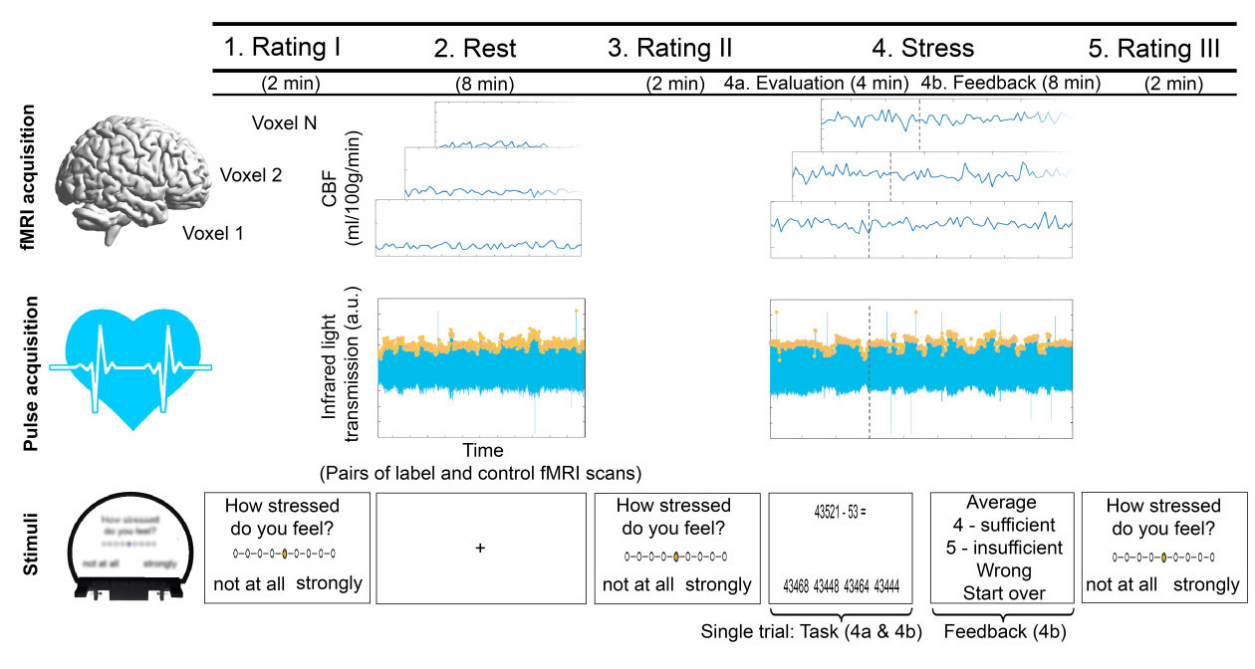
**fMRI stress paradigm**. The paradigm comprised five consecutive stages. In three Rating periods (Stages 1, 3 and 5), the participants reported their current degree of feeling stressed, relaxed, anxious and frustrated. Each of these stages had a duration of 2 min. In the second stage (‘2. Rest’; 8 min), resting ASL fMRI scans and heart rate signals were acquired. The fourth stage (‘4. Stress’; 12 min) comprised the fMRI stress task which was adopted from Weygandt et al. (2016). Pulse data were acquired in parallel to brain activity. For further details, see text.

In Stage 4, the participants were asked to perform a series of subtraction tasks with two operands X and Y depicted in the upper part of a computer screen as fast as possible. In order to solve a task, the participants had to select the single correct answer included in a set of four possible answers depicted in the lower part of the screen with the response box. At the beginning of the paradigm, operand X was equal to 43 521 across all participants. Y ranged from 1 to 99 and was randomly determined across all trials. The stress task was divided into two consecutive stages, i.e. the ‘Evaluation’ stage (4a) implemented to assess the participants’ personal performance capability (4 min) and the ‘Feedback’ stage (4b) included to derive neural and peripheral stress-processing parameters (8 min). In the Evaluation stage, all participants had 8 s time to select an answer across all trials, no feedback was provided but response times were recorded. For trials following correctly solved trials, X was equal to the difference X minus Y in the preceding trial. In case of false/too slow answers, X remained unchanged. The Feedback stage differed from the Evaluation stage in three important points. First, the participants’ performance was rated additionally. Specifically, depending on the difference between the fastest correct response of the participant in the Evaluation stage and the response time in a given trial of the Feedback stage, the trial performance was evaluated in terms of school grades ranging from ‘1—sehr gut’(very good) to ‘5—ungenügend’ (insufficient). Second, the time provided for response selection (which was 8 s at the beginning of the Feedback stage) was decreased (increased) by 10 per cent in case of correct (false) answers in preceding trials. Finally, third, X was reset to 43 521 in case of false/too slow answers. Before the experiment, the participants were told that they will take part in an arithmetic task comprising feedback and that this feedback will evaluate their output in terms of performance markers established in the overall population. After task participation, they were informed that the feedback was computed by relating their trial performance to their performance in the Evaluation stage. Figure 1 illustrates the paradigm.

### MRI sequences

Anatomical brain scans were acquired with a T_1_-weighted 3D-magnetization prepared rapid gradient echo sequence (MP-RAGE; 176 slices; 1 mm isotropic voxels; time of repetition (TR) = 1900 ms; echo time (TE) = 3.03 ms; flip angle (FA) = 9°; field of view (FOV) = 256 mm × 256 mm; matrix size = 256 × 256; 4 min 26 s) and a T_2_-weighted fluid-attenuated inversion recovery sequence (FLAIR; 176 slices; 1 mm isotropic voxels; TR = 6000 ms; TE = 388 ms; TI = 2100 ms; FA = 120°; FOV = 256 mm × 256 mm; matrix size = 256 × 256; 7 min 44 s). Functional scans were measured with a pseudo-continuous ASL EPI sequence^35^ with 22 ascending transversal slices covering the whole brain [slice thickness 5.75 mm (15% inter-slice gap); 3 mm × 3 mm in-slice voxel resolution; TR = 4000 ms; TE = 19 ms; FA = 90°; FOV = 192 × 192 mm^2^; matrix size = 64 × 64; label duration 1.5 s, post-label delay 1.2 s; anterior to posterior phase-encoding direction]. In the Rest condition (8 min), 60 control and 60 label ASL images were acquired, in the Stress condition (12 min) 90 control and 90 label scans. Two spin-echo EPI reference volumes with matching read-out and geometry were acquired in advance to the rest and the stress ASL measurements to facilitate a distortion correction.

### MRI preprocessing

#### Anatomical brain scans

A manual mapping of focal lesions, a tissue segmentation of T_1_-weighted images, as well as a determination of a GM group mask for the fMRI analyses were performed. For details, see Supplementary material.

#### Functional brain scans

The fully automatized fMRI preprocessing pipeline comprised seven steps which were performed with (toolboxes for) SPM12 (Wellcome Trust Centre for Neuroimaging, Institute of Neurology, UCL, London UK—http://www.fil.ion.ucl.ac.uk/spm). In (i), we corrected the raw ASL images for head motion using the ASL toolbox.^36^ In (ii), we used the SPM12 coregistration algorithm to map both spin-echo EPI reference images with opposing phase-encoding direction to the average motion-corrected image determined in (i). In (iii), we used the HySCO toolbox^37^ and both coregistered spin-echo EPI reference images to correct the realigned fMRI images computed in (i) for inhomogeneity of the main magnetic field. In (iv), we linearly coregistered the images determined in (iii) to the high-resolution anatomical T_1_-weighted images. After coregistration, (v) the images were smoothed with a Gaussian kernel (8 mm full width and half maximum). In (vi), we used the ASL toolbox to compute voxel images of the average regional cerebral blood flow (rCBF; ml/100 g/min) for the Rest (2) and the Feedback-Stress stage (4b) based on control–label pairs. Finally, (vii) we used the coregistration parameters determined in the segmentation of anatomical T_1_-weighted scans (see Supplementary material) to register the average rCBF maps of both task stages to the standard space defined by the Montreal Neurological Institute^38^ (voxel size 3 mm × 3 mm × 3 mm). Finally, we identified ASL data of individual participants with insufficient quality separately for both conditions using the framewise displacement (FWD) metric, an established data quality marker evaluating participants’ head (i.e. whole brain) motion.^39^ FWD scores across all participants in individual conditions more extreme than the first (third) quartile—(+) 1.5 × inter-quartile range (IQR) were considered outliers and excluded.

### Computation of stress-related neural network activity

**Figure 2:**
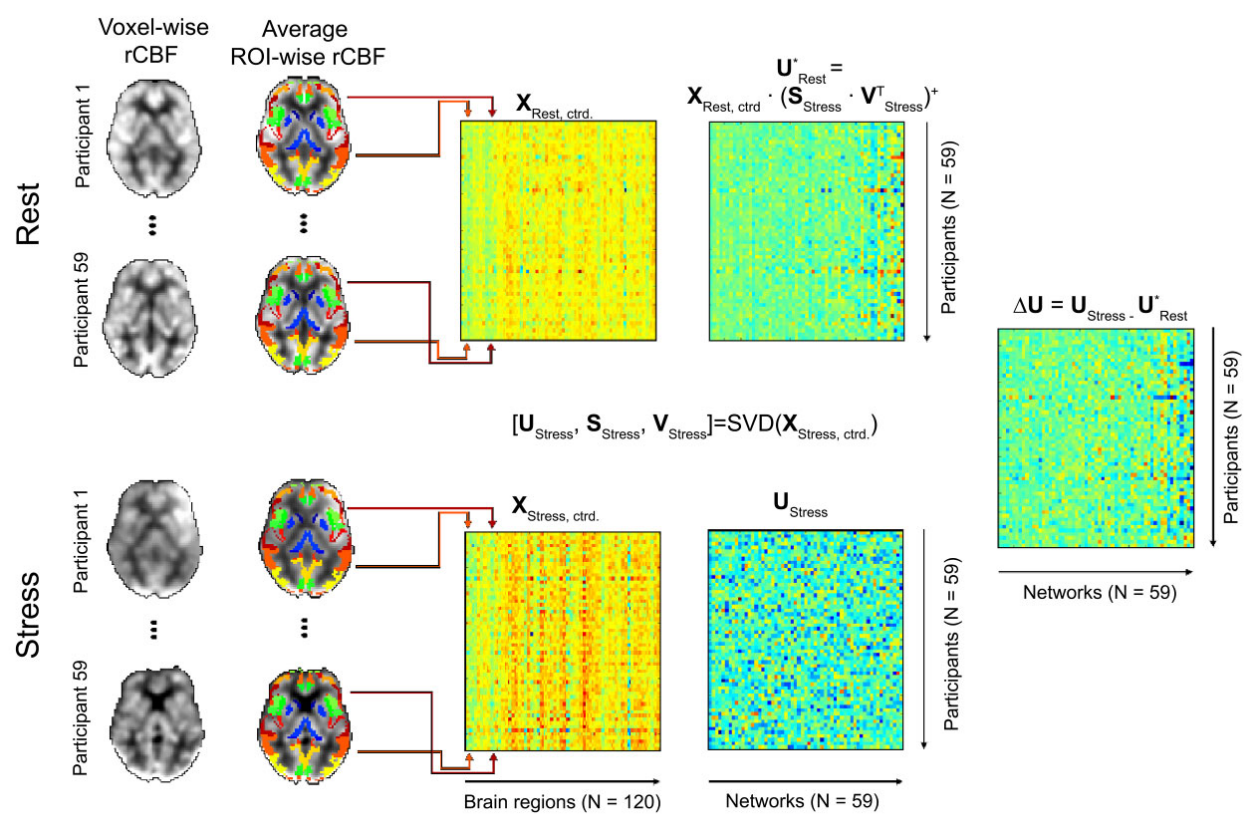
**Illustration of the network activity computation procedure.** The coloured areas in the right column of brain slices highlight specific regions included in the Neuromorphometrics brain atlas (http://Neuromorphometrics.com) and located in the respective slices. SVD was used to decompose a matrix of (manifest, observable) input variables **X**_Stress, ctrd._ into matrices of (latent, unobservable) variables **U**_Stress_, **S**_Stress_ and **V**_Stress._**X**_Stress, ctrd._ comprised the averaged (and centred) rCBF acquired during the stress stage for each participant (one participant per matrix row) and atlas regions (one region per matrix column) computed across all voxels located in a given region included in the atlas and covered by the GM mask. Each column of the component matrix **U**_Stress_ reflects the activity of a given network (one column per network) across participants (one column element per participant). The matrix of Eigenimages **V**_Stress_ reflects the similarity between the averaged and centred regional activity and the network or component activity. Finally, the matrix of singular values **S**_Stress_ reflects the magnitude of the components’ contribution to the variance in the input variables and can be used to compute the explained variance. By computing **X** = **U** × **S** × **V***^T^* it is possible to reconstruct the manifest input data from the latent variables or to map the data from latent component space into manifest regional space, respectively. Utilizing this fact, we calculated the activity of our networks during the resting stage as U*_Rest_ = **X**_Rest_ × (**S**_Stress_ × **V**^T^_Stress_)^+^. Finally, we computed differential stress response network activity as **ΔU** = **U**_Stress_ − U*_Rest_. Please note, that we could not compute **U**_Rest_ via decomposing **X**_Rest, ctrd._, as the covariation among regions (i.e. the FC) during stress is different to that during rest. The figure was adapted from Meyer-Arndt *et al*.^26,76^

We determined stress-induced alterations in FC or neural network activity, respectively, for each participant based on the rCBF maps of those participants for whom non-outlier scans were available for both fMRI conditions (*N*_Total_ = 59; *N*_MS_ = 36; *N*_HP_ = 23) using singular value decomposition (SVD). SVD is a widely established data processing method frequently used in genetic and neuroimaging studies,^40,41^ which is closely related to principal component analysis (PCA; i.e. SVD is one matrix factorization method that can be used for PCA). SVD is well suited to evaluate FC^42^ as it characterizes a large set of input variables (matrix **X** in Fig. 2) by computing (i) few new, mutually independent variables (i.e. ‘components’; matrix **U** in Fig. 2) reflecting the characteristic variation in the input variables and (ii) variables (sometimes referred to as ‘Eigenimages’; matrix **V** in Fig. 2^42^) reflecting the similarity between all components and input variables. Based on these similarities, the input variables can be assigned to groups of functionally connected regions or neural networks, respectively (see below). Each component can be understood as single variable encoding the activity of a corresponding network for each participant with a single number. Moreover, SVD allows computing (iii) the proportion of variance explained in the input variables by the individual components (using matrix **S** in Fig. 2).

In practical terms, we first calculated measures of regional rCBF for each participant by averaging rCBF across all voxels located in a region included in an anatomical atlas and covered by a GM mask (see Fig. 2 for details). This was done for each atlas region and for Stages 2 and 4b separately. Then, we subtracted the average rCBF computed across all regions from all regional signals for each participant and condition (‘centring’). The data computed for stress (stage 4b) were then used as input data for SVD which was employed to determine parameters (i) to (iii) described above. The number of components determined by SVD equals the smaller dimension of the factorized data matrix (spanned by 59 participants and 120 GM regions) and thus corresponded to 59 in this case. Each component reflects the shared characteristic variation in the average rCBF of individual GM brain regions contributing to the corresponding network across participants. Because computing (iii) showed that the first component (explaining the maximum amount of variation in the input variables in SVD) explained less than half of the variation in the input variables (i.e. 45%), we evaluated all 59 components in our subsequent analysis of a differential coupling of brain activity and gene expression in patients and controls (see ‘Differential coupling of brain activity and gene expression in patients and controls’ section). In the next step, we centred the regional rCBF data acquired during Stage 2 and used these data and the Eigenimages determined based on the stress data (i.e. **V**_Stress_) to compute component variables for the Rest condition. Furthermore, we subtracted the component variables for Rest from those for Stress to obtain measures of differential network activity which were then evaluated in subsequent fMRI group analyses. Figure 2 illustrates the procedure. Finally, we employed a ‘Winner-Takes-All’ strategy, to group individual brain regions into neural networks. Specifically, we assumed that a region belonged to a network/component, if the relative contribution of the component’s signal to the regional signal (indicated by its absolute value in **V**_Stress_) was larger than that of any other component.

### Preprocessing of GC-related gene expression data in T cells

We quantified gene expression of four major components of the GC signalling in CD4+ and CD8+ T cells: glucocorticoid receptor (*GR*), FK506-binding protein 4 (*FKBP4*), FK506-binding protein 5 (*FKBP5*) and glucocorticoid-induced leucine zipper (*GILZ*). The GR is the main intracellular receptor for GCs including cortisol and plays a key role in immunoregulation.^43^ FKBP5 acts as a co-chaperon that modulates GR activity and mediates the stress response in the immune system (and other tissues^44^). Upon GC binding of GR, FKBP5 is exchanged for FKBP4, thereby initiating nuclear translocation and downstream transcriptional activity. Finally, GILZ is transcriptionally induced by GR and mediates major anti-inflammatory actions of GCs, particularly in T cells.^45^ To quantify the expression of these eight markers, complementary DNA was amplified using a real-time PCR System. Then, gene expression of the eight markers was normalized to the expression of housekeeping genes and delta cycle threshold (ΔCT) values were calculated by subtracting the mean CT values of the gene of interest from geometric mean of housekeeping genes. For details, e.g. on isolation of peripheral blood mononuclear cells and sorting of CD4+ and CD8+ T cells, RNA isolation, cDNA synthesis and Real-Time Reverse Transcription PCR, see Supplementary material.

Because the eight individual markers were considerably inter-correlated (the average absolute correlation across all 28 marker pairs was 0.25, for four pairs the absolute correlation was above 0.50), we then used the individual markers to compute one characteristic summary marker of GC-related T-cell gene expression to avoid redundant statistical analyses. Specifically, in a first data quality assurance step, we searched for outliers among gene expression data in each of the individual markers (ΔCT values smaller than the first quartile—1.5 × IQR or larger than the third quartile +1.5 × IQR) and retained only the data of those 49 participants (*N*_MS_ = 29; *N*_HP_ = 20) for whom non-outlier data for all eight markers were available. In the next step, we centred the remaining data and performed a SVD based on all eight individual markers as input variables. Because the first component explained 70% of the variance in all eight individual markers, this component was used as a single summary measure of T-cell GC signalling in all subsequent analyses. The variation in the summary marker was most similar to GILZ and least similar to FKBP4 (correlation with CD8 + GILZ: *r* = 0.92; CD4 + GILZ: *r* = 0.79; CD4 + GR: *r* = 0.60; CD8 + FKBP5: *r* = 0.58; CD8 + GR: *r* = 0.55; CD4 + FKBP5: *r* = 0.41; CD4 + FKBP4: *r* = 0.15; CD8 + FKBP4: *r* = −0.08).

### Statistical analyses

#### Psychological, peripheral and neural stress responses

To test the main effects of task stage/stress exposure, group and their interaction on stress response measures [perceived stress, 64 participants; heart rate, 54 participants; activity of 120 regions included in a neuroanatomical atlas and covered by a GM mask (Supplementary material); 59 participants], separate factorial repeated measures analyses were conducted. This was done with linear mixed model (LMM) regression (cf. Weygandt *et al.*^46^) implemented in MATLAB 2014a (MathWorks, Natick, MA, USA). Each LMM included three fixed effects regressors or covariates of interest (CI). One main effect regressor for task stage, one for group and a regressor coding their interaction computed as their element-wise product. Sex, age, disease duration since first signs of multiple sclerosis, task load (Supplementary material), depression (BDI) and an intercept corresponded to the fixed covariates of no interest (CNI). An intercept modelling each participant’s average perceived stress level/heart rate/average rCBF across task stages was included as random nuisance parameter. Perceived stress (task Stages 3 and 5), heart rate (2 and 4b) and rCBF (2 and 4b) averaged across voxels located in individual atlas regions (i.e. columns of **X_Rest_** and **X_Stress_** before centring) served as criterion variables. We determined the probability to obtain the observed *t*-statistics by chance with a permutation method for repeated measures (10 000 within-subject permutations of each CI). A significance threshold for undirected tests of *α* = 0.05 was applied for perceived stress and heart rate. A multiple comparison or family-wise error (FWE) corrected significance threshold for undirected tests of *α* = 4.2 × 10^−4^ (computed with the Bonferroni method; i.e. 0.05/120) was applied for tests of brain responses. Standardized regression coefficients *β* are reported as effect size measures with |*β*| < 0.2 indicates a weak, 0.2 ≤ |*β*| < 0.5 a moderate and |*β*| ≥ 0.5 a strong effect.^47^

#### Differential coupling of brain activity and gene expression in patients and controls

To evaluate whether an altered interplay between central nervous stress processing and immunologic functioning exists in PwMS, we tested in an interaction analysis whether stress-induced neural network activity is differentially linked to T-cell GC signalling in PwMS and HP (i.e. hypothesis H1). Specifically, one factorial analysis was computed with robust regression (bisquare M-estimators implemented in MATLAB) for each of the 59 networks based on the data of the 44 PwMS and HPs (*N*_MS_ = 26; *N*_HP_ = 18) for whom non-outlier fMRI and GC-related expression summary data were available.

Robust regression is much less affected by outliers than standard (i.e. ordinary least square) regression and has consequently been proven to increase the statistical power of tests conducted.^48^ In each analysis, an interaction regressor was computed as the element-wise product of differential network activity (Stress minus Rest) and group was included in the model as CI. In addition, a main effect regressor for group (enabling an estimation of differences in GC-related gene expression between PwMS and HP) and for differential activity, as well as regressors for disease duration, sex, age, task load, depression (BDI) and a constant were also included in the model. The T-cell GC expression summary marker served as criterion. A robust permutation algorithm (10 000 permutations^49^) evaluating the resampling distribution of a Wald (W)-statistic was used for inference which is asymptotically valid even in case predictors and errors are uncorrelated, but not independent (e.g. in heteroscedastic regression). Given that evaluating H1 required conducting multiple (i.e. 59) tests, we report interaction effects significant according to an FWE-corrected threshold for undirected tests of *α*_FWE_ = 0.05 [i.e. *α*_single test_ (0.05)/number of tests (59) × 0.0008]. Again, we report *β* coefficients as effect size measures for the CI.

Importantly, we repeated this analysis by replacing the T-cell GC expression summary marker once with parameters of the average diurnal salivary cortisol levels and once with the daytime decline in salivary cortisol to evaluate a differential coupling between stress-related neural network connectivity and salivary cortisol in PwMS versus HPs. Additionally, we tested group differences in salivary cortisol, and differential associations between the T-cell GC-summary marker and salivary cortisol across groups in supplementary analyses (Supplementary Table 1 and Fig. 1).

#### Brain activity and disease severity

Assuming that analysis of *differential coupling of brain activity and gene expression in patients and controls* would identify an altered interplay between CNS stress processing and immunologic functioning in PwMS, we evaluated whether the activity of the identified network(s) fulfilling H1 is associated with four important multiple sclerosis disease severity outcome markers in patients to evaluate its clinical importance. In particular, we evaluated whether activity of this/these network(s) is associated with GM fraction (H2), T_2_-weighted lesion load (H3), clinical disability (EDSS; H4) and information-processing speed (SDMT; H5). To test hypotheses H2–H5, activity of networks fulfilling the criterion of H1 was entered into robust regression analyses as CI (CNI: disease duration, sex, age, task load, depression and constant) and used to model the patients’ GM fraction, T_2_-weighted lesion load, SDMT and EDSS in independent analyses. Lesion load was log-transformed [i.e. log(number of lesion voxels + 0.001)] before the analysis to account for the skewness in its distribution. Permutation testing (10 000 permutations^49^) was used for inference. In order to test H2–H5, we applied a significance threshold of *α* = 0.05 for undirected tests. No multiple comparison correction was necessary, because (i) specific hypotheses could be derived from the literature for predicting each of the four specific severity markers (H2^21^; H3^8^; H4^20^; H5^29^) and (ii) only a single predictor/CI was used (as only one network was found in testing H1; see below) to model each of them.^50^ Again, we report coefficients *β* as effect size measure for the CI. Additionally, we report the strength of the association between the activity of the network selected for severity prediction based on its differential link to T-cell GC sensitivity across groups (i.e. via testing H1) and the four severity measures relative to the strength of this association computed for all other 58 networks’ activity to estimate the relative clinical importance of altered CNS–immune crosstalk in multiple sclerosis. Finally, we also tested associations between the activity of neural networks showing a differential link between stress-related neural network connectivity and salivary cortisol in PwMS vs. HPs with disease severity in patients as well as direct associations between cortisol and disease severity markers (Supplementary Table 1 and Fig. 1).

#### GC-related T-cell gene expression and disease severity

In a complementary analysis, we tested direct associations between GC-related gene expression in T cells and the four disease severity parameters with robust regression. The gene expression summary marker computed with SVD served as CI, sex, age, disease duration and depression (plus constant) as CNI. All other aspects were as reported for brain activity-based severity prediction.

### Software accessibility

The in-house software used in the study will be made available by the corresponding author without restrictions on request.

### Data availability

Structural MRI (sMRI) images will not be made available due to privacy issues of clinical data. Moreover, all data used in this research were collected subject to the informed consent of the participants. Consequently, access to all other (i.e. non-sMRI) data will be granted by the corresponding author on request only in line with that consent, subject to approval by the project ethics board and under a formal Data Sharing Agreement.

## Results

### Demographic and clinical participant characteristics

Demographic and clinical descriptors of the study sample are provided in Table 1. PwMS and HPs were comparable with regard to age and sex. Six of 39 PwMS were treated with dimethyl fumarate, eight with β-interferons, five with glatiramer acetate, five with fingolimod and four with teriflunomide.

**Table 1.** Demographic and clinical participant characteristics of the 64 participants for whom either high-quality fMRI or gene expression data were available

Group	Sex (f/m)	Age (yrs)	SDMT (#corr. trials)	BDI-I (pts.)	GM (fract.)	T2LL (cm^3^)	EDSS (pts.)
	#	MN SD	MN SD	MN SD	MN SD	MD RG	MD RG
MS	25/14	47.12 11.85	53.85 9.92	9.82 7.47	0.40 0.05	6.35 0.15–52.99	2.50 0–6
HP	17/8	42.10 17.06	59.08 15.35	2.84 2.32	0.44 0.05	0.17 0–8.10	
** **	***χ* ^2^** ***p***	***t*** ***p***	***t*** ***p***	***t*** ***p***	***t*** ***p***	***t*** ^a^ ***p***	** **
** **	0.10 0.749	1.39 0.169	−1.66 0.102	4.52 2.8 × 10^−5^	−2.74 0.008	5.90 1.6 × 10^−7^	

T2LL, lesion load as indicated by T_2_-weighted FLAIR images; f, female; m, male; yrs, years; corr., correct; pts., points; fract., fraction; #, number of cases; MN, mean; MD, median; SD, standard deviation; RG, range.

^a^ Inferential statistics testing group differences in T_2_-weighted lesion load were computed based on log-transformed lesion voxel counts.

### Psychological, peripheral and neural stress responses

**Figure 3:**
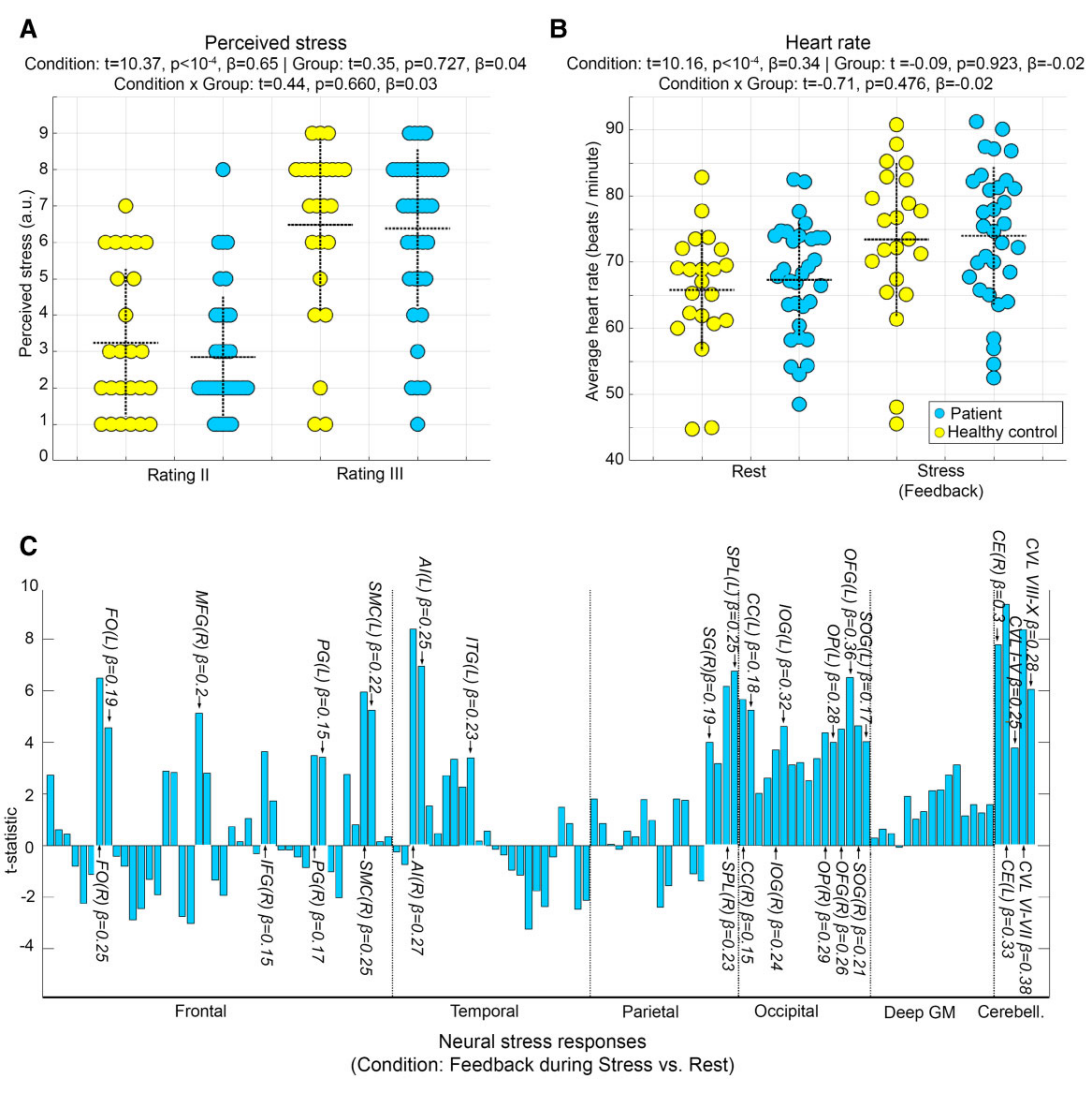
**Psychological, peripheral and neural stress responses.** To test main effects of task stage/stress exposure, group and their interaction on stress response measures, separate factorial repeated measures analyses were conducted with LMM regression for each of the parameters. The dotted horizontal lines in (**A**) and (**B**) show the mean, the vertical lines the standard deviation for the raw values of the depicted parameter and group. The bar graph in (**C**) depicts the *t*-statistic for the main effect of stress exposition/task stage (feedback during Stress vs. Rest) across both groups for all 120 regions included in the neuroanatomical atlas and covered by the GM mask. Regional labels attached to selected bars highlight regions with significant activation differences according to an FWE-corrected threshold for undirected tests of 0.05/120 = 4.2 × 10^−4^. For these regions, we also show the standardized regression coefficients *β* as effect size measures. In particular, significant stress responses (i.e. exclusively positive ones) were observed in AI, calcarine cortex (CC), cerebellum exterior (CE), cerebellar vermal lobules (CVL), frontal operculum (FO), inferior frontal gyrus (IFG), inferior occipital gyrus (IOG) and inferior temporal gyrus (ITG). Moreover, such responses were found in middle frontal gyrus (MFG), occipital fusiform gyrus (OFG), occipital pole (OP), precentral gyrus (PG), supramarginal gyrus (SG), supplementary motor cortex (SMC), superior occipital gyrus (SOG) and superior parietal lobule (SPL)—either of the left (L) or right (R) hemisphere.

The task induced a stress response in perceived stress and heart rate. However, neither main effects of group, nor interaction effects of task stage and group were found. Analyses of neural stress responses revealed several areas with stronger activity during Stress than Rest including right AI and fusiform gyrus (FG; Fig. 3). An interaction of task stage and group was found in only one of 120 regions, i.e. in right parietal operculum (*t* = 4.24, *P* < 10^−4^, *β* = 0.17).

### Differential coupling of brain activity and gene expression in patients and controls

**Figure 4:**
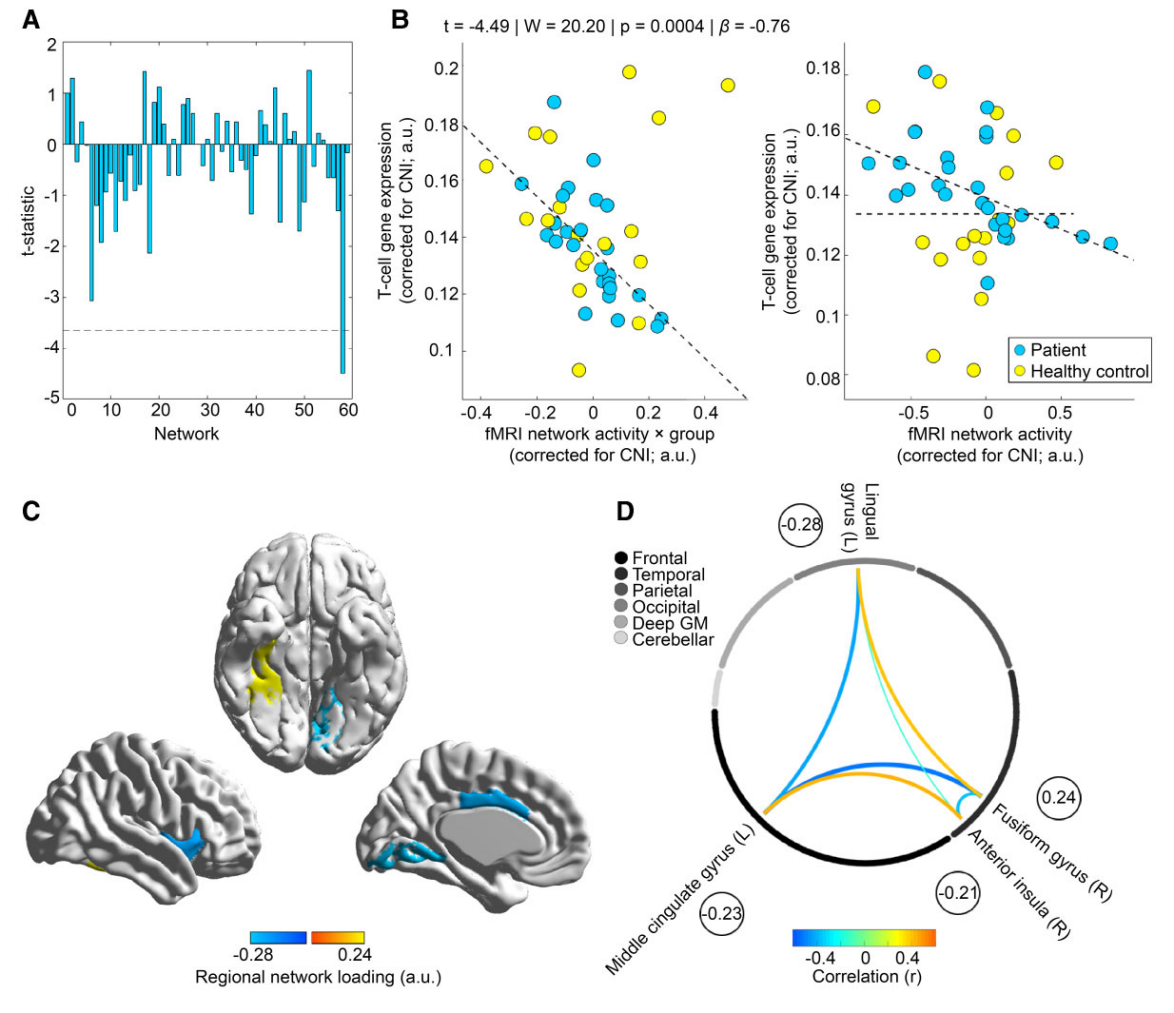
**Differential coupling of neural network activity with GC-related gene expression in T cells across groups.** (**A**) *t*-Statistics for the effect of the interaction between differential stress-activity and group on the GC gene expression summary marker across all 59 neural networks/components. We depict the *t*-statistic instead of the Wald-statistic (used for inference) as the *t*-statistic indicates the directionality of effects. The dashed line depicts the *t*-statistic corresponding to *α*_FWE_ = 0.05 (equal to *α* = 0.05/59 = 0.0008 on a single test level) in an undirected test according to a parametric *t*-distribution for illustrative purposes. (**B**) Differential coupling of GC-related gene expression and stress-induced brain activity in patients and controls for the 58th network showing a significant interaction effect. The left scatter graph depicts the association between the interaction regressor and gene expression and thus reflects the tested effect directly. The right graph depicts the association between brain activity and gene expression for both groups separately as an additional illustration of the interaction. Other than for the model used to compute the fit depicted in the left graph, the models used to compute the two group-specific fits depicted in the right did not include the main effect regressor for group and the interaction regressor for group × network activity. (**C**) Brain areas related to the 58th network/component (see Supplementary material for the network to brain region allocation procedure) and their component loadings. Finally, (**D**) correlations between manifest average voxel rCBF signals for atlas regions belonging to the network across participants. To ease comprehensibility, the encircled numbers again depict the regional component loadings.

The factorial analyses conducted for each of the 59 neural networks to test an altered coupling between stress-triggered network activity and T-cell GC gene expression in PwMS vs. HPs showed a strong interaction effect for a network comprising right AI, right FG, left midcingulate and lingual gyrus on an FWE-corrected significance level (*t* = −4.49, *W* = 20.20, *P* = 0.0004, *β* = −0.76; see Fig. 4). Please note, that for the same network, a moderate main effect of group/a moderate difference in GC-related gene expression of T cells between PwMS and HPs was observed which was significant according to an uncorrected threshold (*t* = 1.93, *W* = 3.74, *P* = 0.048, *β* = 0.47).

### Brain activity and disease severity

**Figure 5:**
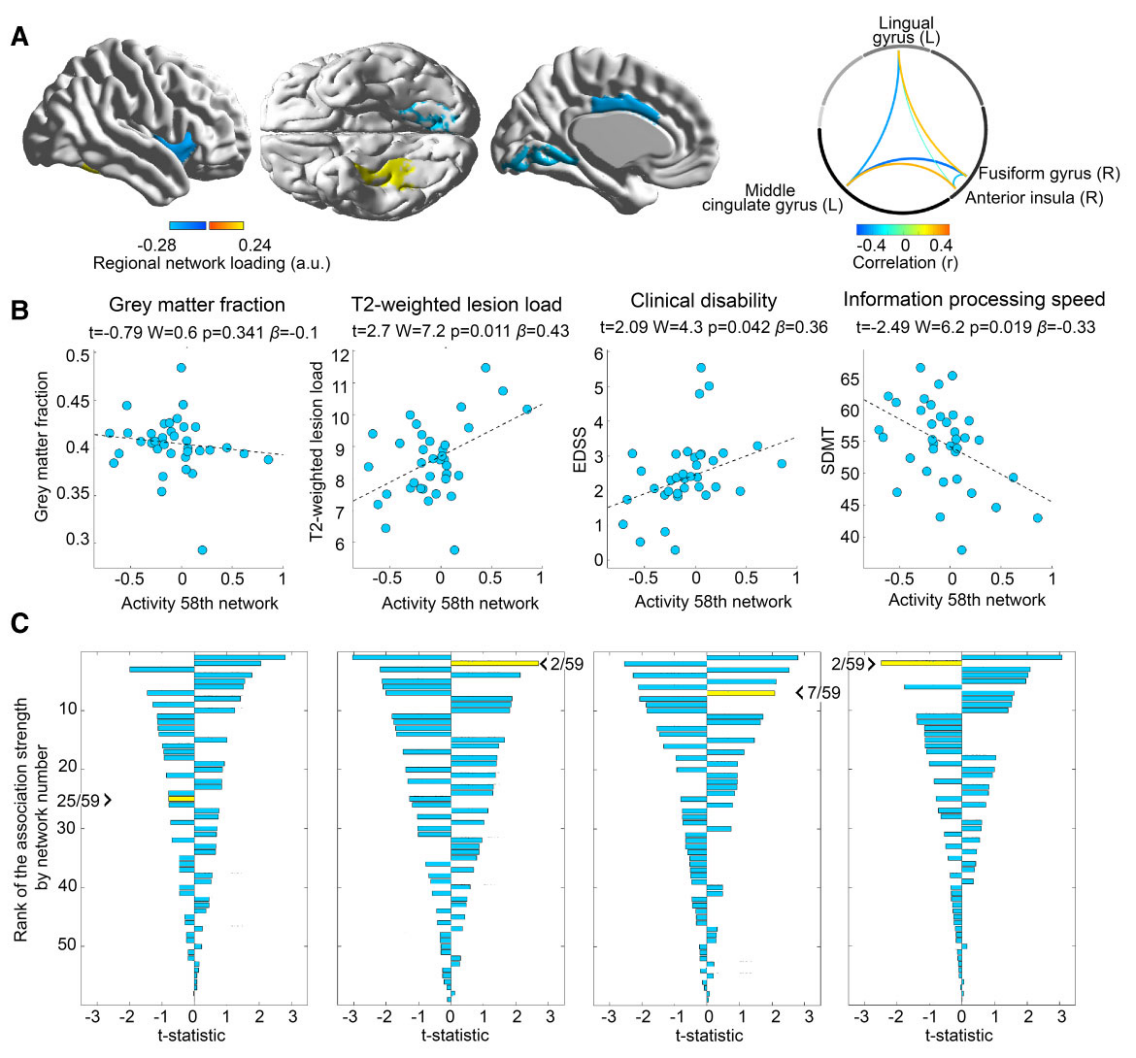
**Disease severity modelling in patients based on brain network activity differentially linked to GC-related gene expression across groups.** (**A**) The topology of and internal connectivity within the 58th network linked to GC-related gene expression in a group-specific fashion to ease the interpretation of the following panels. (**B**) The relation between network activity and each of the four disease severity markers in PwMS. (**C**) The strength of the association between activity of all 59 networks/components and all four severity measures in PwMS, sorted in a descending fashion based on the absolute *t*-statistic to enable an estimation of the relative clinical importance of each network. The network/component whose *t*-statistic is highlighted by the arrowhead is the component selected via testing of H1 (i.e. the 58th network). The rank of this component is additionally illustrated by the first number following the arrowhead.

Brain network activity found in the above analysis showing a differential link to GC-related T-cell gene expression in PwMS vs. HPs showed a significant association with T_2_-weighted lesion load, clinical disability (EDSS), and information-processing speed (SDMT). The relative strength of the association between the 58th network/component (which was selected due to its differential link to T-cell GC sensitivity or testing of H1, respectively) and GM fraction was low as it had the 25th closest association among all 59 networks (i.e. Rank 25 of 59). However, the relative association was strong for the three other measures (second rank for T_2_-weighted lesion and information-processing speed, seventh rank for clinical disability). Figure 5 provides details.

### GC-related T-cell gene expression and disease severity

Despite the size of the negative association between the GC-summary marker and clinical disability/EDSS was moderate, it did not reach statistical significance (*t* = −1.50, *W* = 2.26, *P* = 0.145, *β* = −0.30). Also for the other three parameters, no significant associations were found (GM fraction: *t* = 0.50, *W* = 0.25, *P* = 0.621, *β* = 0.07; T_2_-weighted lesion load: *t* = −0.54, *W* = 0.29 *P* = 0.584, *β* = −0.14; information-processing speed/SDMT: *t* = 0.97, *W* = 0.94, *P* = 0.335, *β* = 0.22).

## Discussion

The link between stress and multiple sclerosis has primarily been investigated in an isolated, discipline-specific fashion. To address this link from an interdisciplinary perspective, we investigated whether CNS stress processing and immune system sensitivity to stress hormones are linked in a disease-specific fashion and whether this link is clinically meaningful. We found that the activity of a network including AI was differentially coupled to T-cell GC signalling in PwMS and HPs and that this network’s activity was simultaneously linked to patients’ lesion volume, clinical disability and information-processing speed.

Preparatory analyses underlined the basic ability of the fMRI task to induce stress, as they revealed a marked stress response on a psychological, peripheral and neural level. Distinct stress-driven neural activity was primarily found in frontal, cerebellar and occipital areas (including FG), as well as AI. This regional pattern strongly overlaps with areas found in similar studies.^10,11^

In our first main analysis, we tested whether CNS stress processing and T-cell GC sensitivity are linked in a disease-specific fashion, as both factors showed a similar pattern of associations with multiple sclerosis severity in prior studies which, however, addressed these associations in an isolated, discipline-specific fashion only. In particular, neuroimaging studies found that blunted CNS stress-processing goes along with higher multiple sclerosis severity^10^ and that neural activity associated with dampened psychological stress experience is linked to higher future brain atrophy.^26^ Neuroendocrine animal research has underlined the importance of (non-blunted) T-cell GC sensitivity for regulation of autoimmune neuroinflammation by demonstrating that T cells downregulate GR expression and dynamically develop functional GC insensitivity during the early phase of experimental autoimmune encephalomyelitis, the animal model of multiple sclerosis.^22^ Consistently, Engler *et al*.^51^ could show that GC sensitivity is reduced in PwMS, Gold *et al*.^22^ that blunted GC sensitivity of T cells in PwMS is associated with neuroinflammation as GC sensitivity was lower in patients with vs. without active MRI lesions. Thus, together, these studies argue that the interplay between neuroendocrine stress processing and T-cell GC sensitivity is altered in multiple sclerosis. Aiming to test a disease-specific CNS–immune interplay that might resemble this altered neuroendocrine-immune one, we tested H1 in our first main analysis and searched for neural networks whose stress task-evoked activity is differentially linked to peripheral T-cell GC signalling in PwMS vs. HPs as a potential indicator of a disease-specific CNS–immune crosstalk. The analysis revealed a strong (*β* = −0.76) differential link for the activity of a neural network comprising right AI, right FG, left midcingulate and lingual gyrus. Inferred from the right graph in Fig. 4B, network activity and T-cell GC sensitivity were linked in PwMS but not in HP.

AI is a key hub between the CNS and the immune system that is sensitive to GC administration^52,53^ and that measures stress-related (peripheral) inflammatory parameters.^54^ Importantly, animal studies revealed that insular cortex can also efferently regulate immunologic processes by demonstrating that lesions induced to the rat insular cortex disrupt the ability to acquire conditioned immunosuppression (i.e. an immune response learned via Pavlovian conditioning^55,56^). Thus, although observational studies do not allow drawing causal inference, these findings on (‘efferent’) conditioned immunosuppression argue that the differential link between stress-related CNS activity and T-cell GC-sensitivity across groups found in our study might potentially indicate an impaired CNS regulation of immune functioning in multiple sclerosis.

Importantly, AI does not only measure and potentially regulate immunological functions, it is also involved in a variety of cognitive and affective processes.^25^ Specifically, insula is the key area for measuring, signalling, encoding and remembering affectively relevant internal bodily states (so-called ‘gut-feelings’^57^), a domain impaired in PwMS.^58,59^ Thus, when considering frequent insula atrophy in PwMS^60^ and their impaired ability to perceive and evaluate peripheral stress signals,^61,62^ one might speculate that altered immunological and affective processes in multiple sclerosis might be connected via AI functioning.

In the second main analysis, we investigated whether the activity of the network identified in testing H1 is associated with disease severity. This analysis supported the majority of the four corresponding hypotheses (H2–H5), as it showed that three of four key severity measures were related. In particular, the network’s activity was linked to lesion load (H3), clinical disability (EDSS; H4) and information-processing speed (SDMT; H5). The effect size of each of the three associations was moderate (lesion load: *β* = 0.43; EDSS: *β* = 0.36; SDMT: *β* = −0.33). These findings show that the altered CNS–immune system crosstalk in multiple sclerosis may be clinically meaningful.

Several aspects of the study might warrant further discussion. First, the inability of our study to reveal a direct link between T-cell GC sensitivity and disease severity might appear surprising on first sight. This fact might become comprehensible, however, when considering that Gold *et al*.^22^ found such an association in PwMS with current inflammatory disease activity whereas the present study exclusively recruited patients on stable immunotherapy and during remission. Thus, this lacking direct link together with the association between three important severity markers on one hand and neural network activity identified by searching for an altered CNS—T-cell GC sensitivity link in PwMS on the other suggests that although T-cell GC signalling might not directly drive disease pathology, it is presumably a part of a larger stress-related mechanism that does.

Similarly, one might wonder why diurnal cortisol did not significantly differ between PwMS and HP and did also not correlate significantly with clinical disability (see Supplementary Table 1 and Fig. 1) although it did in other studies.^63^ Some of these differences might be attributable to differences in the composition of groups between studies. For example, our study did not comprise secondary-progressive MS patients—and cross-sectional associations between EDSS and salivary cortisol were restricted to such patients.^63^ Having said that, our analyses are in line with previous findings of (i) a differential link between cortisol and brain activity across groups for a network comprising both frontal poles and (ii) a link between salivary cortisol and network activity in HP but not PwMS (Supplementary Fig.1H, J) as an association between of frontal pole activity and cortisol in HP was also previously reported.^12,64^

Another point might relate to the question of whether receiving the diagnosis of multiple sclerosis, a severe neurological disease, or subsequent structural alterations might drive differences in central stress-responsivity between patients and controls and whether these differences underly the findings made in the study. Although we agree that structural alterations might lead to alterations in central stress processing, no differences in regional central stress processing between PwMS and HPs were found in the corresponding analysis conducted in this study (e.g. on psychological, peripheral and neural stress responses). Moreover, missing differences in regional neural stress responses between PwMS and HPs are consistent with findings made in our recent study^10^ employing voxel-wise analyses based on the same task and an independent data set. Despite these points, however, replication and extension of these findings in longitudinal studies and/or randomized controlled trials (e.g. applying stress management interventions; see Mohr *et al.*^8^ and Grossman *et al.*^65^) will be needed to further strengthen the confidence in a link between psychological stress and multiple sclerosis.

Additionally, one might wonder whether DMTs applied might have influenced GC-related gene expression in T cells. Human studies addressing this question for the DMTs used in this study, however, did not report significant changes in gene expression for our genes of interest *GR*, *FKBP5*, *FKBP4* or *GILZ*.^66–69^ Moreover, PwMS taking steroid treatment in a period of 4 weeks preceding a potential study participation were not included in the study.

It should be noted that our assays focused on T-cell gene expression as measured by quantitative PCR (qPCR) and we did not include measures of protein levels of these targets. It has been shown that mRNA expression measured by qPCR closely reflects protein levels (e.g. GR,^70^ FKBP4,^71^ FKBP5,^72^ GILZ^73^). Moreover, analyses of the transcription of GC target genes can add important information as responsiveness of GC-inducible genes such as *GILZ* also depends on chromatin accessibility of the regulatory regions (enhancers and promoters). Thus, our approach is a composite measure that captures GC signalling on various levels (including epigenetic regulation) and we, therefore, believe that our choice of mRNA targets provides an excellent estimate of the GC signalling within each cell population studied. Having said that, future studies should expand these read-outs by adding analyses on the protein level as well as functionally probing GC sensitivity.

A final aspect that could be discussed is that the 58th of 59 fMRI networks/components was relevant in this study (i.e. a component that explains only a small fraction in the variation of the input data), as some authors proposed the heuristic that only components explaining a lot of variation can be useful in follow-up regression analyses.^74^ However, this heuristic was disproved by Jolliffe^75^ almost as early as it was formulated by providing several simple examples showing that and why this is not true.

In conclusion, our study shows that stress-related CNS functioning is linked to T-cell stress hormone sensitivity in a disease-specific fashion and simultaneously related to disease severity in multiple sclerosis. Thus, it might have helped to increase our knowledge on factors contributing to the importance of psychological stress for multiple sclerosis reported in large epidemiological studies^4^ and clinical observation^5–7^ as well as treatment studies.^8^

## Supplementary Material

fcac086_Supplementary_Data

## Abbreviations

AI =: anterior insula
ANS =: autonomic nervous system
ASL =: arterial spin labelling
BDI =: Beck-Depression Inventory
CI =: covariates of interest
CNI =: covariates of no interest
DMT =: disease-modifying therapy
EDSS =: Expanded Disability Status Scale
FC =: functional connectivity
FWE =: family-wise error
FG =: fusiform gyrus
FLAIR =: fluid-attenuated inversion recovery sequence
FWD =: framewise displacement
GC =: glucocorticoid
GM =: grey matter
HP =: healthy person
HPA =: hypothalamic–pituitary–adrenal axis
LMM =: linear mixed model
PCA =: principal component analysis
PwMS =: persons with multiple sclerosis
rCBF =: regional cerebral blood flow
SDMT =: Symbol Digit Modalities Test
SVD =: singular value decomposition
