# Supplementary material for: Central stress processing, T-cell responsivity to stress hormones and disease severity in multiple sclerosis
Source: Brain Commun. 2022 Apr 4;4(2):fcac086. doi: 10.1093/braincomms/fcac086 (PMC9014535; doi:10.1093/braincomms/fcac086)
Supplement: fcac086_Supplementary_Data [file fcac086_supplementary_data.docx]

**Central stress processing, T cell responsivity to stress hormones,
and disease severity in multiple sclerosis**

**Supplementary material**

**Central stress processing, T cell responsivity to stress hormones,
and disease severity in multiple sclerosis**

**Abbreviated title: Stress processing and multiple sclerosis**

Jelena Brasanac^1,2,3,4,5^, Stefan Hetzer^6^, Susanna Asseyer^1,3,4,5^, Joseph Kuchling^1,7,8^,

Judith Bellmann-Strobl^1,3,4,5^, Kristin Ritter^2^, Stefanie Gamradt^2^,
Michael Scheel^1,9^, John-Dylan Haynes^1,6,10^, Alexander U. Brandt^1,3,4,5,11^,

Friedemann Paul^1,3,4, 5,7§^, Stefan M. Gold ^2,12,13,§^, and Martin Weygandt ^1,3,4,5,§,*^

^1^ Charité – Universitätsmedizin Berlin, corporate member of Freie Universität Berlin, Humboldt-Universität zu Berlin, and Berlin Institute of Health, NeuroCure Clinical Research Center, 10117 Berlin, Germany

^2^ Charité – Universitätsmedizin Berlin, corporate member of Freie Universität Berlin, Humboldt-Universität zu Berlin, and Berlin Institute of Health, Department of Psychiatry and Psychotherapy, Campus Benjamin Franklin, 12203 Berlin, Germany

^3^Experimental and Clinical Research Center, a cooperation between the Max Delbrück Center for Molecular Medicine in the Helmholtz Association and Charité Universitätsmedizin Berlin, Germany

^4^Charité – Universitätsmedizin Berlin, corporate member of Freie Universität Berlin and Humboldt-Universität zu Berlin, Experimental and Clinical Research Center, Lindenberger Weg 80, 13125 Berlin, Germany

^5^Max Delbrück Center for Molecular Medicine in the Helmholtz Association (MDC), Berlin, Germany

^6^ Charité – Universitätsmedizin Berlin, corporate member of Freie Universität Berlin, Humboldt-Universität zu Berlin, and Berlin Institute of Health, Berlin Center for Advanced Neuroimaging, 10117 Berlin, Germany

^7^ Charité – Universitätsmedizin Berlin, corporate member of Freie Universität Berlin, Humboldt-Universität zu Berlin, and Berlin Institute of Health, Department of Neurology, 10117 Berlin, Germany

^8^ Berlin Institute of Health (BIH), 10178 Berlin, Germany

^9^Charité – Universitätsmedizin Berlin, corporate member of Freie Universität Berlin, Humboldt-Universität zu Berlin, and Berlin Institute of Health, Department of Neuroradiology, 10117 Berlin, Germany

^10^ Charité – Universitätsmedizin Berlin, corporate Member of Freie Universität Berlin, Humboldt-Universität zu Berlin, Berlin Institute of Health, Bernstein Center for Computational Neuroscience, 10117, Berlin, Germany

^11^ Department of Neurology, University of California, Irvine, CA, USA

^12^ Charité – Universitätsmedizin Berlin, corporate member of Freie Universität Berlin, Humboldt-Universität zu Berlin, and Berlin Institute of Health, Department of Psychosomatic Medicine, 10117 Berlin, Germany

^13^ Institute of Neuroimmunology and Multiple Sclerosis (INIMS), Center for Molecular Neurobiology Hamburg, Universitätsklinikum Hamburg-Eppendorf, 20251 Hamburg, Germany

Key words: multiple sclerosis, psychological stress, T cell glucocorticoid signaling, functional brain connectivity, Arterial-Spin-Labeling functional MRI.

§ These authors contributed equally

* Corresponding author.

Charité – Universitätsmedizin Berlin, Charitéplatz 1, 10117 Berlin, Germany.

Email: martin.weygandt@charite.de.

Materials and methods

Processing of heart rate data

A single characteristic heart rate parameter was computed for each participant and each experimental fMRI stage based on raw pulse signals measured at participants’ toes with a standard pulse oximeter (sampling frequency 50 Hz) included as supplementary device in the MRI scanner hardware. (Please note that the quality of heart rate signals assessed at participants toes has been proven to be comparable to that of signals assessed at their fingers^1^.) This was done in two steps. Specifically, we first computed one Fourier analysis based on the raw pulse signals of a given participant measured during the rest stage and one for the data measured during the feedback period in the stress stage with the FFT algorithm implemented in Matlab. In a second step, we used the Matlab FIT algorithm to fit a unimodal gaussian function to the amplitude spectrum computed by FFT for both periods and took the frequency with the maximal fitted amplitude as single characteristic heart rate parameter for this participant and experimental stage. Two preprocessing steps were applied to the amplitude spectra prior to gaussian modeling to stabilize the fit. First, frequencies below 30 Hz and above 150 Hz were discarded to constrain the fit to the physiologically meaningful frequency spectrum. Second, amplitudes in a frequency window from 47.6 to 48.4 Hz as well as in a window ranging from 95.6 to 96.4 Hz were discarded due a technical artefact generated in these frequency ranges by the pulse oximeter.

Measurement and processing of diurnal salivary cortisol

Levels of salivary cortisol were assessed in the morning (after waking up) and in the evening at 21h on two consecutive days prior to MRI scan. On the day of clinical examination, participants were given instructions and salivettes (Sarstedt, Germany) for saliva collection at home. Samples were returned by the participants on the day of the MRI scan, centrifuged for 5min at 1000 x g, aliquoted and stored at -20 °C until analysis. The cortisol level was measured using enzyme-linked immunosorbent assay (ELISA)(IBL, Germany) following the manufacturer’s instructions.

We computed one parameter for the average salivary cortisol level across the day and one for the decline in daytime salivary cortisol using multiple regression for each participant separately. A regression model was generated including one regressor coding for the daytime of each of the four salivary cortisol measurements and centered it to model the decline in daytime salivary cortisol. An intercept was included to model the average salivary cortisol level across the day. The resulting two regression coefficients determined for the model were used as measure for the average salivary cortisol level across the day and as measure for the decline in daytime salivary cortisol. After these parameters were determined, we computed one parameter for the average salivary cortisol level across the day and one for the decline in daytime salivary cortisol using multiple regression for each participant separately. In particular, we generated a regression model including one regressor coding for the daytime of each of the four salivary cortisol measurements and centered it to model the decline in daytime salivary cortisol. An intercept was included to model the average salivary cortisol level across the day. The resulting two regression coefficients determined for the model were used as measure for the average salivary cortisol level across the day and as measure for the decline in daytime salivary cortisol.

Computation of task load

To determine a measure of cognitive or task load during the stress condition for each participant, we computed the average time between the onsets of trials or the average intertrial interval respectively during the Feedback period within the Stress stage.

Preprocessing of GC-related gene expression data in T cells

Isolation of Peripheral Blood Mononuclear Cells

On the morning of the clinical assessment, a venous blood sample (around 70 ml) was collected from participants in heparinized vials and processed within 2h. Specifically, Peripheral Blood Mononuclear Cells (PBMCs) were isolated using density gradient centrifugation following established operating procedures^2^. In particular, blood was first diluted in phosphate-buffered saline (PBS) (1:1), 35ml of diluted blood were very carefully layered on top of 15ml of Biocoll density medium (Biochrom, Berlin, Germany) in a 50ml conical tube. Tubes were centrifugated at 870 x g for 30min (brakes off). The mononuclear cell layer from the interphase was harvested and washed two times for 10 min in cold PBS. Pelleted PBMCs were resuspended in RPMI-1640 (Gibco, ThermoFisher Scientific, Berlin, Germany) supplemented with 25% heat-inactivated fetal bovine serum (FBS) (Biochrom, Berlin, Germany) and 10% dimethylsulfoxide (Applichem GmbH, Darmstadt, Germany) for cryopreservation. After counting, cells were placed in 1.5ml tubes (Eppendorf, Hamburg, Germany) at the concentration of 10 million cells/ml and put in Mr. Frosty freezing container (Sigma-Aldrich, St. Louis, USA) for slow overnight cooling in a -80 **°**C freezer. The next day, frozen tubes were transferred to a long-term liquid nitrogen storage tank (-196 **°**C) where they stayed until further analysis.

Sorting of CD4+ and CD8+ T cells, RNA Isolation, cDNA Synthesis, and Real-Time Reverse Transcription PCR

CD4+ and CD8+ T cells populations were sorted using magnetic-activated cell sorting (MACS) that separates cells based on surface antigens. Frozen tubes with PBMCs were thawed in a warm water bath (37 **°**C) for 1-2 minutes and washed in warmed RPMI-1640 medium containing 10% FBS for 6 min at 250 × g. Subsequently, cell sorting was performed using CD4 and CD8 MicroBeads (Miltenyi Biotec, Bergisch Gladbach, Germany) following manufacturer’s instructions. Per 10 million cells, 80µl of MACS buffer (phosphate-buffered saline, 0.5% BSA and 2mM EDTA) and 20µl of CD4 MicroBeads (Miltenyi Biotec, Bergisch Gladbach, Germany) were added and incubated for 15min at 4**°**C in the dark. Cells were washed with MACS buffer for 5min at 350 × g (4 **°**C) and resuspended in 500µl MACS buffer. Next, CD4+ T cells were separated on MACS LS columns (Miltenyi Biotec, Bergisch Gladbach, Germany). In the next step, the CD4- negative fraction was used for sorting CD8+ T cells, following a similar protocol: Cells were labeled with CD8 MicroBeads (Miltenyi Biotec, Bergisch Gladbach, Germany), incubated and separated on MACS MS columns (Miltenyi Biotec, Bergisch Gladbach, Germany), and resuspended in 500µl MACS buffer in the end. Purity checks of cell fractions were conducted by flow cytometry (FACSCanto II, BD Biosciences, New Jersey, USA). For the samples in this study, the following purity coefficients were obtained: CD4+ T cells 91.67% + 6.74 SD (0.89 SEM), CD8+ T cells 95.15% + 5.73 SD (0.76 SEM).

Total RNA was isolated from CD4+ and CD8+ T cell fractions using Qiagen RNeasy Plus Mini Kit (Qiagen, Hilden, Germany) following the manufacturer’s instructions. Concentration and purity were assessed by NanoDrop spectrophotometer (NanoDrop 2000c, Thermo Fisher Scientific, Berlin, Germany). Using RevertAid H Minus First Strand cDNA Synthesis Kit (Thermo Fisher Scientific, Berlin, Germany) complementary DNA (cDNA) was then transcribed from RNA according to the manufacturer’s instructions and stored at -20 **°**C.

Amplification of cDNA was done on a StepOne real-time PCR System (Thermo Fisher Scientific, Berlin, Germany) using TaqMan Gene Expression Assays (Thermo Fisher Scientific, Berlin, Germany) for GR (Hs00353740_m1), FKBP5 (Hs01561006_m1), FKBP4 (Hs00427038_g1) and GILZ (Hs00608272_m1). All real-time PCR reactions were performed in triplicates. Gene expression was normalized to the geometric mean of two housekeeping genes: TATA Box Binding Protein (TBP; Hs00427620_m1) and Importin 8 (IPO8; Hs00183533_m1). These were selected because they have previously been demonstrated to be reliable gene references for gene expression analysis in human T cells^3^. Delta cycle threshold (ΔCT) values were calculated by subtracting mean CT values of the gene of interest from geometric mean of housekeeping genes. All subsequent statistical analyses were done based on these ΔCT values.

MRI preprocessing

Anatomical brain scans

Preprocessing of high-resolution anatomical MRI data comprised three steps.

*Manual mapping of focal hyperintense lesions*

In the first, a manual mapping of focal hyperintense lesions was performed by experienced raters from the group of Prof. Paul under supervision of a neuroradiologist (M. S.) with the software ITK-SNAP (<http://www.itksnap.org>) and based on participants’ FLAIR images. Based on the resulting dichotomous lesion voxel maps (each voxel was either classified as lesion or non-lesion voxel), we computed participants’ T2-weighted whole brain lesion load. Given that the voxel resolution of FLAIR images and thus also the lesion voxel maps was 1 mm^4^, the number lesion voxels identified by the manual mapping procedure simultaneously corresponded to the lesion volume in mm^3^. Finally, in order to account for the frequent non-normality of T2-weighted whole brain lesion volume data, we performed a log-transform of these whole brain lesion volume data^4^. The resulting data were evaluated in all corresponding statistical group analyses.

*Tissue segmentation of T1-weighted images*

In the second, probability tissue maps for GM, white matter, and cerebro-spinal fluid were determined based on MP-RAGE images with the combined spatial normalization and segmentation algorithm implemented in SPM12 (Wellcome Trust Centre for Neuroimaging, Institute of Neurology, UCL, London UK ­ <http://www.fil.ion.ucl.ac.uk/spm>). Voxel coordinates located in lesioned tissue as indicated by the lesion masks generated in the first step (that were coregistered to the T1-weighted images before the segmentation) were excluded from that procedure. This step yielded voxel-wise tissue probability maps in native image space and in the anatomical standard space defined by the MNI^5^. Images computed in the standard space were corrected for potential effects of local deformations applied during spatial normalization. Additionally, the lesion masks coregistered to the T1-weighted images were coregistered to the MNI space for computation of a GM group mask (see below) using the spatial mapping parameters determined in the combined normalization and segmentation procedure. Voxel tissue probability maps computed in native image space were used to compute the GM tissue fractions for each participant by dividing the number of GM voxels by the sum of all intracranial voxels.

*Determination of a GM group mask*

In the third step, a GM group mask for the fMRI analyses was computed. For this purpose, the average tissue probability was computed across all participants for each voxel and each of the three tissue types separately. If a voxels’ average probability for GM was larger than for the two other tissues and not located in lesioned tissue in a single participant (indicated by spatially normalized lesion masks), this coordinate was included in the GM group mask. To account for potential partial voluming effects, the six voxels located in direct vicinity to the lesion coordinates determined across all participants (i.e. with an Euclidean distance of no more than voxel) were removed from the mask.

Statistical analyses

Group differences in salivary cortisol

Differences in salivary cortisol for both cortisol markers (i.e., diurnal average and decrease per hour) between groups (and all other effects related to salivary cortisol) were tested using robust linear regression. Disease duration, sex, age, task load, depression (BDI) and a constant were included in the model as CNI. Undirected tests were conducted.

Associations between salivary cortisol and disease severity in patients

In these analyses, disease duration, sex, age, task load, depression (BDI) and a constant were included in the model as CNI. Undirected tests were conducted.

Differential associations between the T-cell GC summary marker and salivary cortisol across groups

An interaction regressor computed as the element-wise product of the T-cell GC summary marker and group was included in the model as CI. A main effect regressor for group and for the T-cell GC summary marker, as well as regressors for disease duration, sex, age, task load, BDI and a constant were included in the model as CNI. Undirected tests were conducted.

Differential coupling of brain activity and diurnal salivary cortisol in patients and controls

This analysis was conducted exactly as analysis *Differential coupling of brain activity and gene expression in patients and controls* in the main text except for the fact that we conducted it once with the regression coefficients computed as measure of average diurnal salivary cortisol as criterion and once with the coefficients computed for temporal decline in diurnal salivary cortisol as criterion. Undirected tests were conducted.

Link between disease severity and brain activity showing a differential link to diurnal salivary cortisol across groups

This analysis was conducted exactly as analysis  *Brain activity and disease severity* in the main text except for the fact that we conducted it using neural network activity putatively showing a differential coupling of brain activity and diurnal salivary cortisol (and not T cell GC gene expression) in patients and controls as predictor for disease severity in patients. Undirected tests were conducted.

Results

Statistical analyses

Group differences in salivary cortisol

Neither the average diurnal salivary cortisol (t = 1.03, p = 0.297, *β* = 0.28, Supplementary figure 1A) nor the decline in diurnal cortisol per hour (t = -1.29, p = 0.206, *β* = -0.33, Supplementary Figure 1B) differed between groups.

Associations between salivary cortisol and disease severity in patients

Supplementary table 1 below reports the (lacking) associations between average diurnal salivary cortisol and the four disease severity measures in patients as well as between decrease in salivary cortisol per hour and the four measures. Supplementary Figure 1C and 1D further illustrate the findings.

**Supplementary Table 1.** Salivary cortisol and disease severity in multiple sclerosis.

|  | Average diurnal salivary cortisol | | | Decrease in salivary cortisol per hour | | |
| --- | --- | --- | --- | --- | --- | --- |
|  | t | p | *β* | t | p | *β* |
| GM fraction | -0.36 | 0.723 | -0.05 | 0.22 | 0.832 | 0.03 |
| T2-weighted lesion load | -0.10 | 0.924 | -0.02 | -0.18 | 0.859 | -0.04 |
| EDSS | 1.01 | 0.324 | 0.20 | -0.74 | 0.470 | -0.15 |
| SDMT | 0.55 | 0.589 | 0.09 | -0.72 | 0.478 | -0.12 |

Differential associations between the T-cell GC summary marker and salivary cortisol across groups

Associations between the T-cell GC summary marker and salivary cortisol did not differ across groups (average diurnal salivary cortisol: t = 1.50, p = 0.129, *β* = 0.27, Supplementary Figure 1E; decline in diurnal cortisol per hour: t = -1.62, p = 0.112, *β* = -0.28, Supplementary Figure 1F)

Differential coupling of brain activity and diurnal salivary cortisol in patients and controls

This analysis revealed that activity of the 7^th^ neural network (i.e., SVD-component) showed a significantly different association to the decline in diurnal cortisol per hour in PwMS than HPs (t = -4.11, p = 0.0002, *β* = -0.40; Supplementary Figure1H, I). Specifically, whereas the decline in diurnal cortisol per hour seemed to respond strongly to activity of this network in HPs, it appeared unrelated in PwMS (Supplementary Figure 1I). The network comprised the frontal poles in the left and right hemisphere (Supplementary Figure 1J).


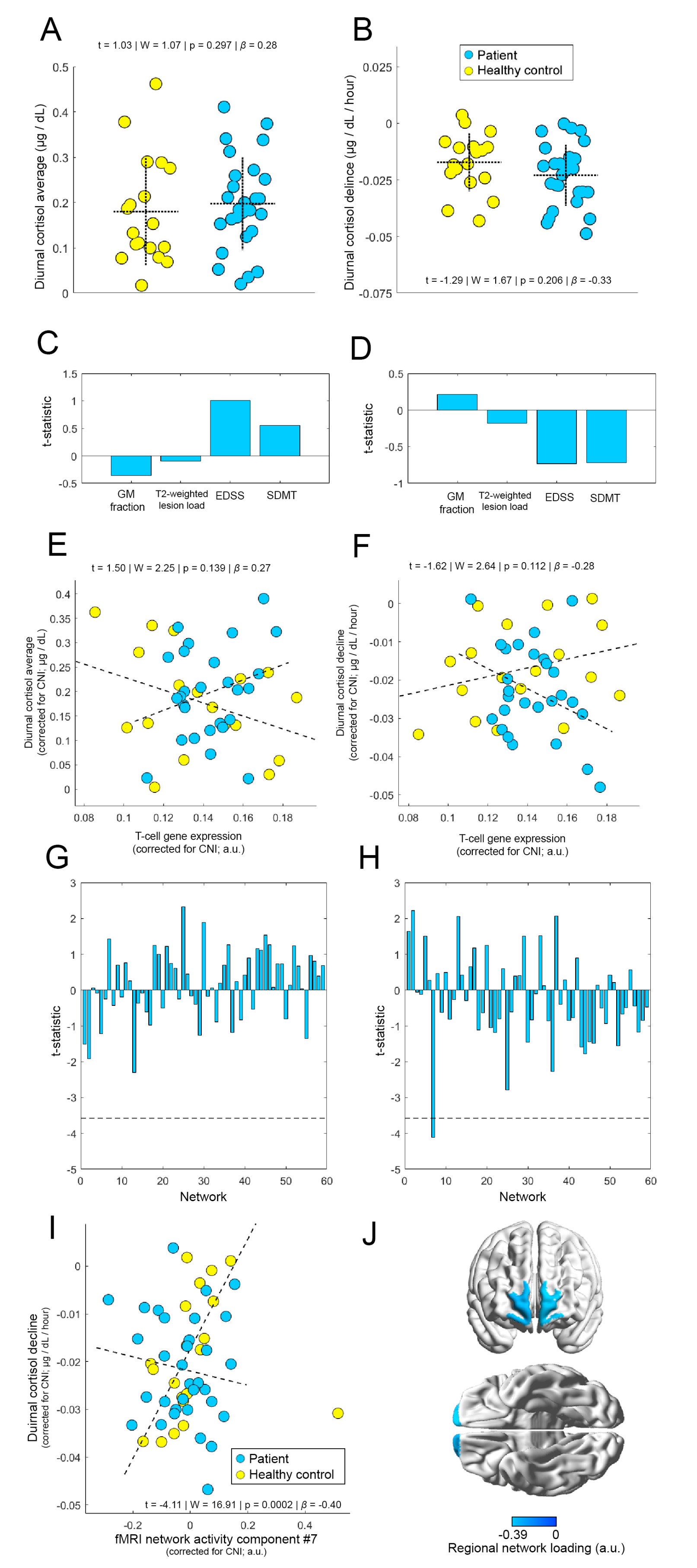


**Supplementary Figure 1. Salivary cortisol.** Group differences in (**A**) the diurnal average salivary cortisol and (**B**) the decrease per hour. The dotted horizontal lines in (**A**) and (**B**) depict the mean, the dotted vertical line the standard deviation for the respective parameter. Associations between (**C**) the diurnal average salivary cortisol and the four disease severity parameters in patients. (**D**) shows these associations in patients for the decrease per hour. (**F**) tests differential associations between the T cell GC summary marker across groups for the diurnal average salivary cortisol and (**G**) depicts that for the decrease per hour. (**G**) shows differential associations between the activity of the 59 neural networks across groups with the diurnal average salivary cortisol and (**H**) depicts that for the decrease per hour. The dashed line depicts the FWE-corrected significance threshold (α_FWE_ = 0.05, undirected). This analysis showed that activity of the 7^th^ neural network was differentially linked across groups, which is additionally illustrated in (**I**). Finally, (**J**) illustrates the two regions belonging to this network: left and right frontal pole.

Link between disease severity and brain activity showing a differential link to diurnal salivary cortisol across groups

This analysis showed that none of the four disease severity markers was significantly related to activity of the 7^th^ neural network in patients (GM fraction: t = -0.64, p = 0.444, *β* = -0.08; T2-weighted lesion load: t = 1.03, p = 0.312, *β* = 0.18; EDSS: t = 0.28, p = 0.764, *β* = 0.05; SDMT: t = -0.102, p = 0.915, *β* = -0.015; see also Fig. 5C in the main text for the weak relative association strength).

References

1.Hinkelbein J, Hose D, Fiedler F. Comparison of three different sensor sites for pulse oximetry. *Int J Intensive Care*. 2005;12:159-63.

2.Hasselmann H, Gamradt S, Taenzer A, et al. Pro-inflammatory Monocyte Phenotype and Cell-Specific Steroid Signaling Alterations in Unmedicated Patients with Major Depressive Disorder. *Front Immunol*. 2018;92693.

3.Ledderose, C., Heyn, J., Limbeck, E. and Kreth, S. Selection of reliable reference genes for quantitative real-time PCR in human T cells and neutrophils. *BMC Research Notes*. 2011;4:427.

4.Chard DT, Brex PA, Ciccarelli O, et al. The longitudinal relation between brain lesion load and atrophy in multiple sclerosis: a 14 year follow up study. *J Neurol Neurosurg Psychiatry*. 2003;74:1551-1554.

5. Tzourio-Mazoyer N, Landeau B, Papathanassiou D, et al. Automated anatomical labeling of activations in SPM using a macroscopic anatomical parcellation of the MNI MRI single-subject brain. *Neuroimage.* 2002;15:273–89.
